# German Psychology
*"Allgemeine Zeitschrift für Psychiatrie und Psychischgerichtliche Medicin; achter Band; hefte drittes, und viertes." Herausgegeben von HerrnDamerow, Fleming, und Röller.


**Published:** 1852-10-01

**Authors:** 


### Art. II.—GERMAN PSYCHOLOGY.*

Two parts of the eighth volume of this valuable journal are now before us. They contain a sufficient amount of important and interesting matter to afford satisfactory evidence that Psychological Medicine occupies much of the attention of German Physicians, while the continued support and assistance accorded to the conductors of the "Zeitschrift fur Psy-

* "Allgemeine Zeitschrift für Psychiatrie und Psychischgerichtliche Medicin; achter Band; hefte drittes, und viertes." Herausgegeben von HerrnDamerow, Fleming, und Röller.

CHIATRIE" by many contributors, demonstrate that its editors have correctly apprehended and rightly met the occasion for a Journal of Psychological Medicine, as the medium of the publication of matters having relation to diseases of the mind, and the pathology of the nervous system.

We shall on the present occasion first lay before our readers a brief analysis of the contents of each part.

The contents, then, of the third part of this eighth volume are:—

*The common Sensibility of the Brain.* By Dr. Fr. Nasse.—This is a short paper in which the author points out the impossibility of forming the diagnosis of the seat of disease within the cranium by the localization of the pain attendant thereon.

*The disordered Connexion of Thought with voluntary Movements through the Influence of the Brain.* By Dr. Fr. Nasse.—In this paper the author's object is to apply physiological knowledge of parts of the brain to the diagnosis of the seat of those diseases which manifest a derangement of voluntary movements. The surface of the hemispheres (by which we suppose the author means the *hemispherical ganglion* of Solly), he observes are concerned in the processes of thought and intellectual operations; and by the connexion of these external parts with the deeper portions, *e. g.*, the corpus striatum and the optic thalamus, the functions of which are perception and the exercise of the will,* the influence of the mind upon voluntary movements is exerted. The author points out the exalted state of the hemispherical surface in certain states of insanity in which confusion of thought is exhibited in incoherency of speech; he traces an analogy in the convulsions which sometimes accompany this state, with that of incipient intoxication, of sleep-walking, and talking, and of chorea. In these instances the excitement of the outer parts of the brain is transferred to the central organs of perception and volition. The same line of reasoning is employed under the opposite condition of dulness of the intellectual operations and disinclination to movement which is witnessed in melancholy insanity. The rambling garrulity of idiots and demented persons is also referred by the author to irregular excitement of the before-mentioned parts of the brain, through their connexion with the organs of speech. In catalepsy, thoughts are formed, but the muscles are not concordantly excited to action; in ecstasy, the same condition is inferred to exist in a higher degree, the hemispherical ganglion being active while the motor influences are in abeyance. In epilepsy, the author argues that both the superficial and the central parts of the brain are disordered, as seen in the simultaneous occurrence of coma and convulsion. Dr. Nasse adds that this point of diagnosis acquires

* Todd, in *Med. Gaz.*, 1849, p. 815.

importance from the consideration that a lesion of voluntary motion may have its cause external to the brain, in the spinal cord, in the nerves distributed over the body, or in the muscles themselves, at the same time it is more probable that an extensive lesion of this function must have its seat in the parts leading to the hemispheres.

*Self-delusion in its psycho-pathological and medico-forensic relations.* By Dr. Schuster.—In this place the author concludes at some length the observations contained in the preceding part of the journal.

*On the employment of Opium in Mental Disease and some allied conditions.* By Dr. Friedr. Engelken, of Oberneuland.—It will be interesting to our readers to learn the views of our German brethren upon a practical point which has particularly engaged attention in England. The author introduces his remarks by a few general observations upon the empirical misuse of medicines; and in the next place gives an historical sketch of his subject. The use of opium for mental maladies, among the ancients, Dr. Engelken observes is very doubtful, since we have no written record thereof, and their theories of this class of diseases would be opposed thereto. The first distinct mention of its employment in mental diseases, he informs us, is to be found at the beginning of the eighteenth century by Dr. Cullen. By Tralles and Wepfer it was given in increasing doses until sleep was produced. The views of Reil, the author remarks, coincide with those which guide the administration of opium in insanity, by the best practitioners of the present day, as seen by the following quotation from that writer's treatise on fever:—"In asthenic mania with erethism, not proceeding from any material (*organic?*) cause, opium administered in full doses, from one to four grains, is of most essential service; it diminishes excitement, quiets the undue action of the brain, and causes sleep. Further, it is of great utility in cerebral disturbance from cold, accompanied with pain and spasms."

The writers whose names we next meet with are those of Fribourg, Pargeter, Chiarugi, and Friedrich; the latter ranges the authorities into two classes, those opposed to and those in favour of the use of opium in insanity; among the former he enumerates Prichard, Haslam, Hasper, Cox, Neville; in the latter, Chiarugi, Reil, Burrows, have not, according to the author, sufficiently indicated the contra-indications of its employment. Friedrich's indications for its use are excitement in a depressed state of the cerebral vitality, and the necessity for the production of a soothed state of the mind. The influence of Brown's views, Dr. Engelken remarks, was to hinder the use of opium in the cases now spoken of, and despite the commendations of Sydenham, its use was prohibited, and the treatment of mental disease was, by so much, prejudiced during part of the present century.

Opium, Dr. Engelken observes, was formerly regarded as the common representative of all narcotics, but later researches have shown that its narcotic properties are unlike those of others of the class, while in value it surpasses all others. The mode of action of opium advocated by the author is that of those physiologists who consider it to have a twofold action, one local, on the nerves of the stomach, the other, remotely, on the nervous centres, by absorption into the blood.

In illustration of the effects of opium, the author quotes Reineke's description (in Blumenbach's *Medic. Bibl. Bd.* 11. § 340) of the Persian and other oriental opium eaters, and observes thereon, that we may thence learn that opium may be administered in larger doses, and for a longer continuance than is generally admitted. In support of this opinion, Dr. Engelken cites several of his own cases, in which from one to three grains had been given with benefit once or twice a day, for periods of three or four years, and in one instance, with two short intervals, for a period of twenty years. We may observe, however, upon the supposed beneficial result in these instances, that time must be regarded as an important element in the cure. Dr. Engelken has often administered this remedy for three months, and longer, in different forms of mental disease, without having perceived any ill effects to have resulted; on the contrary, the appetite has improved, the entire frame has been benefited, besides the marked and decisive amelioration of the mental malady. It has seldom been found requisite to give so large a dose as four grains. Medium doses have usually been combined with other means; regardless of the primary excitement, the use of the drug has been persevered in, limited to once or twice in the twenty-four hours.

The general influence of opium, the author divides into positive and negative, determined by the amount of the dose, thus he describes small (*e. g.* half grain) doses as producing augmentation of the rapidity of the circulation, and of the quantity of the secretions;—if the dose be raised to a grain, or a grain and a half, the actions of the brain are increased, with diminished susceptibility to external impressions. Thoughts are developed more rapidly and with greater clearness, the association of ideas is more varied, and imagination more active. A larger dose, *e. g.* from three to ten grains, or more, produces the well-known phenomena of stupor, &c. The author further observes, that taken altogether, the primary and secondary effects of opium are exerted upon the nervous system, producing in general a diminution of excitability, and an increase in the capability of action in the mental endowments.

Dr. Engelken enumerates the following as the chief points to be considered in the employment of opium:—the bodily constitution, the nature of the disease, the contra-indications for its employment, the history of the disease.

The changes which time has introduced into our manners, customs, habits, &c. &c., have had their influence in producing a greater development of certain feelings and passions, with their corresponding morbid conditions, and by their frequent repetition, induce a preponderance of the nervous constitution. Opium, the author states, is more suitable for those forms of hypochondriasis which most nearly approach to melancholia, as the former can in many cases be more closely traced to disorder of the visceral ganglia than of the brain itself, to which the morbid state applies more strictly in melancholia. In neither form, however, does the author look for great benefit from its use. In general insanity, the utility of this medicine is observed where there is a degree of excitement, its continued use is then frequently of much service. In mania its employment is not required in the earlier stages, which are marked by more or less of inflammatory, or sub-inflammatory action. This state having been in some measure subdued, the author administers opium in doses of one or two grains, gradually increased to four or six grains, combined with calomel and digitalis. Warm baths and corresponding regimen being enforced at the same time.

Puerperal mania the author recognises as a disease of nervous excitement, with debility occurring in a peculiar inflammatory state, and a form of mania in which the best effects are obtained from opium. In idiotcy and dementia the author finds opium of no service.

Dr. Engelken recognises an asthenic and a sthenic form of delirium tremens, the former in his experience being more frequently met with—nine out of eleven cases. He administers opium in doses of from two to four grains, morning and evening, with or without digitalis.

Chorea is a form of nervous disease in which the author also states that he has witnessed the most decided benefit from opium. He gives it in increasing doses of from one quarter of a grain to one grain, with children of from ten to fifteen years of age, and continues its use for from two to eight weeks.

The contra-indications for the use of opium in mental disease mentioned by the author, are much the same as in other cases; *e. g.*—1. In insanity depending upon inflammation, with or without synochal fever. Besides inflammation of the brain, of which delirium is a symptom, there are many other distinct forms of disease which in the acute stages are attended by delirium, and for which an antiphlogistic rather than a sedative treatment is adapted. 2. In congestive conditions in the arterial (*sanguine?*) temperament opium is injurious; whereas, on the contrary, in the nervous and venous (*lymphatic?*) temperament, opium will, in the majority of cases, remove the congestion, especially when the exciting cause is to be sought in violent mental emotion.

With disease of the mind occurring in the asthenic state, the greatest caution is required in the administration of opium.

With regard to the repetition of the doses of opium, Dr. Engelken points out that this must be determined by the constitution of the patient, and the effects of the previous administration.

The author also observes upon the error of regarding all narcotics as equally useful in mental diseases; and repeats his remark, that they are not to be regarded, as they were formerly, specifics for insanity.

The next contribution consists of a *Report from the public Asylum at Sonnenstein, in Saxony;* and forms the continuation of a similar document published in the third volume of the journal. The report shows that the number of admissions has been steadily on the increase during the last twenty years. The population having also during the same period, 1830—1850, increased from 1,500,000 to 1,900,000. The augmentation of these admissions Dr. Klotz, the reporter, attributes less to a real increase in the frequency of the disease, than to a diminution of the objections to reception into asylums. The number in the asylum in 1850 was 757; the discharges 380; the mortality 9 per cent. Many points of local interest are contained in this report.

The succeeding paper is *On the Parisian Asylums,* by Dr. Droste; on which we need not detain our readers, as they have found the same information in our own pages.

*In the department of " Literature,"* we meet with an analysis of the contents of our own journal for 1849; also, notices of M. Cheneau's "*Recherches sur le Traitement de l'Epilepsie,*" and of Richter's "*Organon der Physiologischen Therapie.*"

Bibliography; or, short notices of original works and contributions to periodicals, by German, French, and English authors; with a Miscellany, or selection of extracts from other journals, conclude this number, which we now leave, to proceed to the fourth part of the volume. The first article is a paper on *Progressive General Paralysis,* by Dr. Sholz, surgeon to the asylum at Hall, in the Tyrol.

During the period 1841—1850, there were received into the institution 257 men and 181 women; among these were 28 cases of general paralysis in 22 men, 6 women; about $\frac{1}{16}$ of the admissions. The ages of these were as follows:—One 25 years, sixteen from 30 to 40 years, nine from 40 to 50, two from 50 to 60 years of age. Hereditary disposition existed in five cases.

Dr. Sholz thus states the results of dissection in 11 of the above cases. In five there were more or less distinct evidences of chronic hydrocephalus, effusion into the ventricles; in one there was general softening of the brain; in one, softening of the gray substance; in one, inflammatory exudation on the dura mater; in one, partial softening of the brain and spinal cord, with serous effusion; in one, there was induration of the posterior lobes, cerebellum, and pons, with great fulness of dark blood and slight serous effusion; in one, tubercular disease of the lungs only could be found, and it was doubtful whether the structure of the brain was in any degree altered.

In Dr. Stolz's private practice, he had met with softening of the grey substance and effusion in one case; medullary sarcoma, with softening of brain and cerebellum in another; and in a third, a fibrous cyst, producing softening, congestion, and effusion.

The author observes with reference to the pathology of general paralysis, that the dissections he has recorded agree with the observations of other writers in referring it not to any single, but to various morbid conditions of the brain, and that the immediate cause of death may always be found in changes of structure in the brain or nervous centres, although it may seem to have resulted from disease of more distant organs, as in the case of acute tubercular inflammation of the lungs. These proximate causes consist in such changes in the brain, or a part thereof, as shall interfere with or suspend its functions, although not speedily fatal, and are dependent upon more remote organic changes. The progress of the disease not being so much dependent upon the rapidity or extent of the interference with the functions of the brain, but by the interruption to the influence of the nervous centres upon the organic processes by which the dissolution of the individual becomes inevitable before a long period has elapsed. In simple insanity the case is very different. In this case a mere functional derangement exists, without complete destruction of the functions of the brain, while the remainder of the organism is never so directly or to such a degree implicated in the danger.

Dr. Stolz has never seen an undoubted case of general paralysis undergo a cure, either by the powers of nature, or by the aid of medical science.

The author concludes his observations with a record of cases.

*Pathological exposition of the characteristics of the different Cerebral Organs and their Functions.* By Dr. Bergmann.—This contribution comprises a series of dissections in fatal cases of disease of the brain, &c., and are valuable and interesting, but do not demand further notice on the present occasion, as they were fully dwelt upon in our last number.

*History of the case of a "Mother in an Asylum."* By Dr. Karl Hergt—possessing some general interest, but appears to be invested with importance from local circumstances.

*Opinion upon the State of Mind of an Incendiary.* By Dr. Heinrich Ellinger.—In this case the history of the individual showed several previous attacks of insanity, and leaves no doubt of the existence of mental derangement.

*On the English non-restraint System, and its employment in Germany.*—Under this head we meet with two contributions, the one from Dr.

Fr. Stimmel, the other from Dr. Guggenbühl, who is known to English readers by his letter to Lord Shaftesbury, in behalf of Cretins.

Dr. Stimmel observes that precise information upon the subject of non-restraint seems to be wanting among German psychological physicians, and therefore he contributes his short notice.

When on a scientific tour in England, he had particular opportunities of observing this system, and sums up what he there saw as consisting in the fact of four or five assistants forcibly holding down a violent maniac patient until he was exhausted by his struggles, and sank into a quiet state. If these means failed after about a quarter of an hour, the patient was transferred to the padded cell, of which the writer enters into a full description.

Having been impressed with this apparent triumph of humanity, the author eagerly introduced the plan into his own asylum; and, from the observation of the results in seven cases, concludes that in milder cases of excitement the padded cell is not only a more humane form of treatment, but also more speedily terminates the paroxysm; while, in the more violent forms it is not only not serviceable, but tends to augment the excitement, and gives the disease a more formidable and fully developed character. For this reason Dr. Stimmel has returned to the old system of treatment, and warns others against being led away by the delusion.

Dr. Guggenbühl speaks, however, in terms of approbation of the results of the non-restraint system as carried out in England, and adduces the good effects of the humane system in the treatment of idiots and cretins.

*The Statistics and Management of the Provincial Institution for Lunatics, at Halle.* By Dr. Damerow.—The statistics here given present the numbers admitted and discharged during six years, from the opening of the institution in 1844 up to December, 1850, viz., admitted males, 467; females, 306; total, 773. Discharged, males, 302; females, 209; total, 511. Remaining, December, 1850, males, 165; females, 97; total, 262. Of these there were curable, males, 79; females, 34; total, 83. Incurable, males, 116; females, 63; total, 179.

A full staff of officers and attendants is described, and a statement is also given of the amount of work done, and stock in clothing, &c., manufactured by the patients.

Out of 149 deaths, the following are among the pathological conditions:—apoplexy, 14; convulsions, 4; softening of the brain, 4; water on the brain, 1; paralysis, 44; acute delirium, 3; phthisis, 36; œdema of the lungs, 4; gangrene of the lungs, 2; pneumonia, 5; anasarca, 8; pyæmia, 4; pseudo-erysipelas, 2; cholera, 8; marasmus, 2.

Hereditary tendency among the 773 cases was shown in 187, or about one-fourth.

In the *literary department* of this number of the journal we find notices of Szafkowski's work on the medico-legal and psychological relations of hallucinations; Fleming's report of the lunatic asylum of Mecklenburg-Schwerin; Wunderlich's Hand-book of Pathology and Therapeutics; Henle's Manual of Rational Pathology; Engel's Treatise on the Osseous Structures of the Human Face; Niepec's Treatise on Goitre and Cretinism.

A bibliographical record of new works and important papers follows. The number concludes, like its predecessors, with miscellaneous matters possessing direct interest for those whose practice engages their attention towards psychological studies, and to whom we would commend the perusal of the *Allgémeine Zeitschrift für Psychiatrie.*

